# Harnessing Surface Instabilities for Functional Materials: Mechanics, Morphology, and Emerging Applications

**DOI:** 10.1007/s40820-026-02162-3

**Published:** 2026-04-16

**Authors:** Qiuting Zhang, Yunli Li, Ruifeng Zhang, Enfang Wang, Ye Xu, Yujie Ke, Xi Chen, Gaojian Lin

**Affiliations:** 1 School of Mechanical Engineering & Automation, Beihang University, Beijing, 100191 People’s Republic of China; 2 Wu Jieh Yee School of Interdisciplinary Studies, Lingnan University, Tuen Mun, 999077 Hong Kong SAR People’s Republic of China; 3 Institute of Particle Science and Technology, Northeastern University, Shenyang, 110000 People’s Republic of China

**Keywords:** Surface instabilities, Functional materials, Stretchable devices, Tunable wettability, Biomedical engineering

## Abstract

Surface instabilities, such as wrinkling, folding, and creasing, have transcended their traditional perception as mechanical failures to emerge as a powerful and versatile paradigm for engineering functional surface morphologies in soft materials. This review comprehensively examines the mechanics, fabrication, and rapidly expanding applications of these instability-driven patterns. This review first elucidates the fundamental principles governing the formation of various instability modes, stemming from classical model of thin film–substrate system, and discusses advanced strategies for achieving precise morphological control, including hierarchical and spatially organized structures. Then the core of this review highlights the transformative impact of these tailored surface topographies across diverse fields. Key applications explored include the development of highly sensitive and stretchable electronic skins (E-skins), energy-harvesting triboelectric nanogenerators, deformable optoelectronic devices, physically unclonable features for advanced optical encryption and anti-counterfeiting, engineering surfaces with dynamically tunable wettability, and biomimetic constructs for biomedical engineering and artificial tissues. Finally, a forward-looking perspective on the challenges and future opportunities in this vibrant field was provided, emphasizing the potential of integrating stimuli-responsive materials, computational design, and artificial intelligence to develop the next generation of intelligent, adaptive, and multifunctional surfaces

## Highlights

- Surface instabilities like wrinkling are recast as a versatile design paradigm for creating functional morphologies in soft materials, moving beyond their traditional view as mechanical failures.
- Precise control over hierarchical and spatially organized instability patterns enables tailored properties for advanced applications in electronics, optics, and biomedicine.
- Key emerging applications include highly sensitive E-skins, stretchable batteries and light-emitting diodes, physically unclonable anti-counterfeiting features, and surfaces with dynamically tunable wettability.

## Graphical abstract

**Figure:**
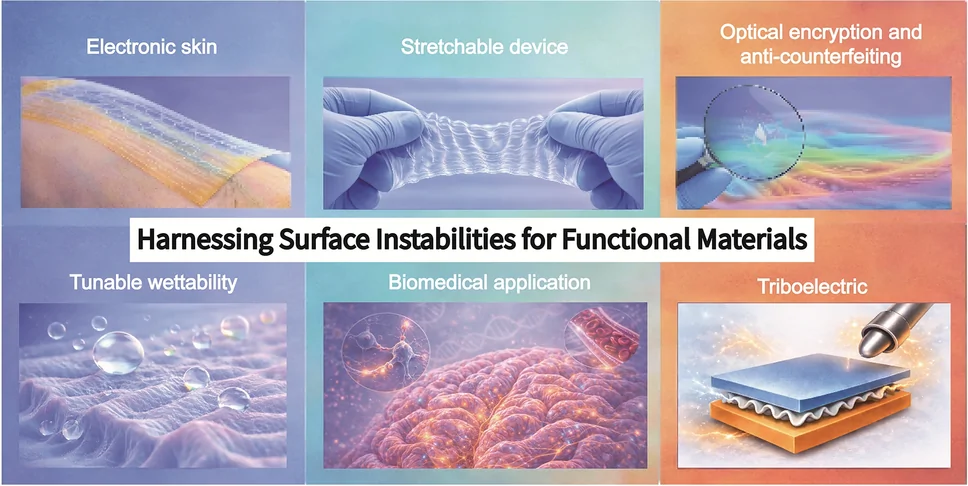

## Introduction

Surface instabilities such as wrinkling, creasing, and folding have become an effective means of producing micro- and nanoscale patterns in soft materials [1–3]. Rather than being considered a form of mechanical failure, wrinkling is now treated as a controllable mechanical response that can be used to construct functional surface morphologies [4]. These instabilities generally result from elastic mismatch between a thin film and a compliant substrate under compression, and the resulting patterns are governed by elastic energy minimization [5–8]. Natural systems offer abundant examples of such phenomena, where surface instabilities play vital roles in growth and adaptation. The convoluted structures of the cerebral cortex, the periodic ridges of fingerprints, and the wrinkled surfaces of raisins and walnuts [9, 10] all originate from mechanical instabilities developed during biological or physical processes. Similar features also occur in skin, leaves, and epithelial tissues as a consequence of internal stresses or differential growth [11]. These structures are usually well regulated rather than random, providing specific advantages such as larger surface area, improved flexibility, or enhanced transport capacity [12].

Insights from these natural cases have inspired biomimetic strategies in materials research, where instability-driven designs are employed to realize controllable and multifunctional surfaces [13–15]. The diversity in surface instability morphologies—from isotropic labyrinths to directional ridges and multilevel folds—endows these structures with unique mechanical, optical, and interfacial functions beyond conventional designs [16–18]. Their ability to respond to external stimuli such as strain, humidity [19], temperature [20, 21], light [22, 23], or electric fields [24] further enables reversible control of surface morphology, making them suitable for adaptive and reconfigurable systems [25, 26]. Alternatively, micro- and nanoscale surface patterns can be fabricated using conventional top-down approaches, such as photolithography, nanoimprint lithography, laser patterning, and mold-based replication techniques [27, 28]. While these methods offer high geometric precision and design freedom, they typically require sophisticated instrumentation, rigid templates, and multistep processing and are often limited in scalability, cost efficiency, or compatibility with soft and stretchable substrates. Moreover, precisely controlling feature wavelength, amplitude, and spatial distribution across curved or flexible substrates remains challenging with these top-down or mold-dependent techniques. In contrast, surface instability-driven approaches harness intrinsic mechanical interactions within material systems to spontaneously generate ordered or hierarchical morphologies over large areas [29, 30], thereby providing distinct advantages that are often inaccessible to conventional fabrication methods, particularly in realizing dynamic and adaptive behaviors [31–33].

Research in this field has expanded rapidly in the past two decades. The understanding of surface instability mechanics has evolved from simple sinusoidal waves to complex hierarchical and anisotropic structures with tunable wavelength, amplitude, and orientation [34]. Such progress has facilitated a variety of practical applications. In optoelectronic systems, wrinkled films improve the mechanical reliability and flexibility of light-emitting diodes (LEDs) and stretchable conductors. In biomedical engineering, dynamic wrinkled surfaces have been applied in antifouling coatings, cell-culture templates, drug delivery systems, and artificial tissues [35]. Similar approaches have also been extended to smart windows, anti-counterfeiting technologies, and water-repellent surfaces inspired by natural wetting states [36–41].

**Fig. 1:**
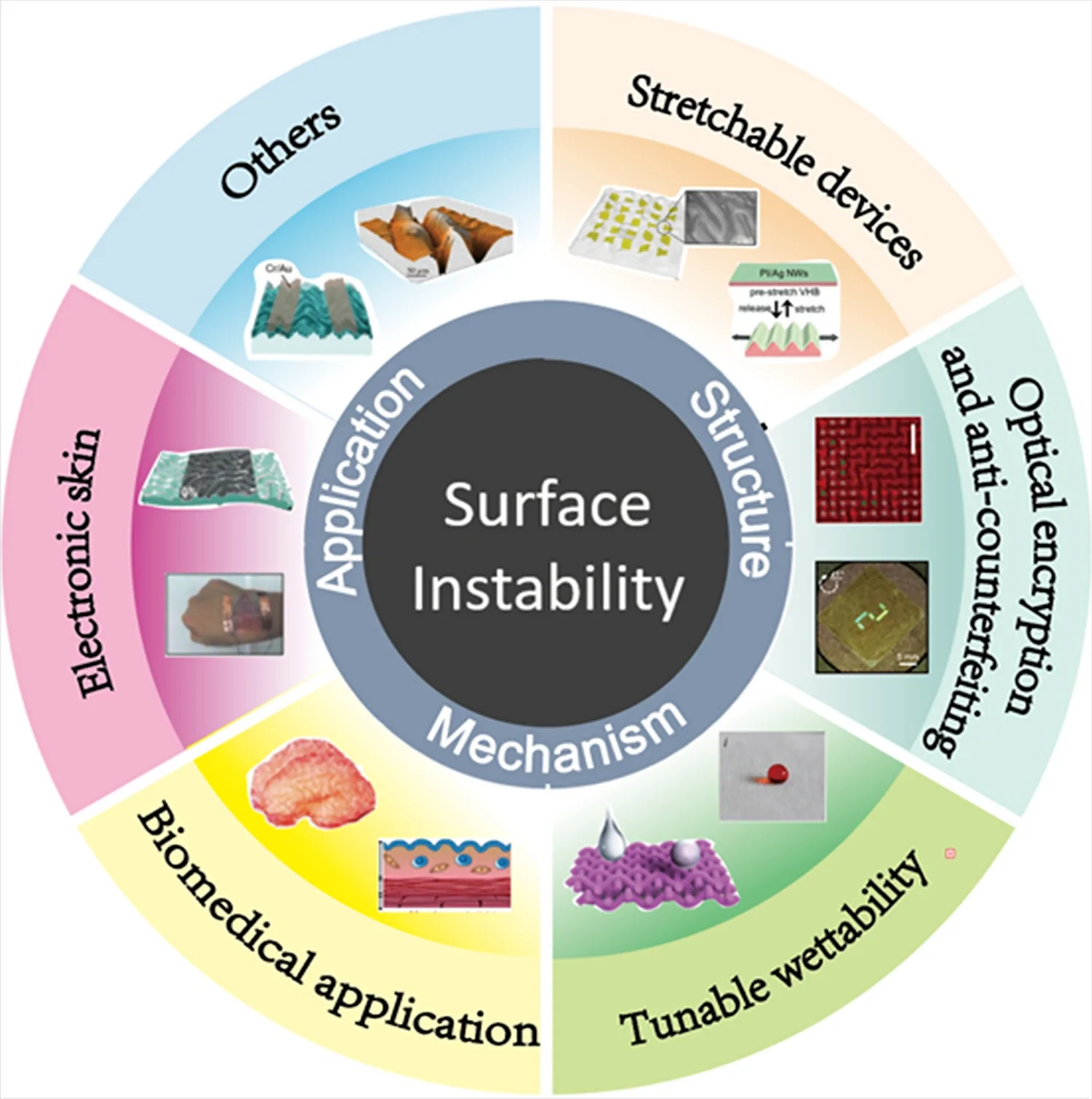
Schematic overview of surface instability–enabled structures and applications. The central panel highlights surface instability as a unifying concept linking mechanism, structure, and application. Surrounding sectors illustrate representative application domains, including stretchable devices, optical encryption and anti-counterfeiting, tunable wettability, biomedical applications, electronic skin, and other emerging areas. Across these domains, diverse instability modes such as wrinkling, folding, creasing, and delamination-induced buckling enable programmable surface morphologies and multifunctional performance through tailored material design and fabrication strategies

This review focuses on the fundamental principles and fabrication strategies of surface instabilities, as well as their emerging applications (Fig. 1). In comparison with existing reviews on wrinkling-, folding-, and creasing-based structures [42], the present review distinguishes itself in several key aspects. At a fundamental level, this review systematically integrates multimode and cross-scale control strategies for surface instabilities. Beyond classical wrinkling phenomena, it provides an in-depth analysis of the underlying mechanisms and morphological distinctions among diverse instability modes including folding, creasing, and delamination-induced buckling and critically evaluates their respective application regimes. From a fabrication and design perspective, the review highlights advanced strategies for constructing hierarchical wrinkling architectures and spatially organized patterns, with particular emphasis on morphological programmability spanning from the nanoscale to the microscale which has not been comprehensively addressed in prior literature. From an application-oriented standpoint, strong emphasis is placed on emerging and frontier applications, showcasing recent advances in stimuli-responsive surfaces for dynamically tunable wettability, optical encryption and anti-counterfeiting, and biomimetic interfaces for biomedical engineering. At the system-integration level, this review advocates an integrated “mechanics–materials–devices” design paradigm, systematically outlining the complete pathway from theoretical modeling and fabrication strategies to functional integration and device-level implementation [14, 43–47].

The overall objective is to provide a clear framework and perspective for the development of instability-driven surfaces with tunable and multifunctional characteristics. Section 2 presents a concise overview of the mechanics and modeling of classical surface instability phenomena, such as wrinkling, folding, and creasing. Understanding the fundamental modeling principles of surface instabilities helps to elucidate the material preparation processes discussed in the subsequent section. Section 3 highlights the emerging applications of surface instabilities across various fields, including electronic skin (E-skin), stretchable devices, optical encryption and anti-counterfeiting, tunable wettability, and biomedical engineering. Finally, Sect. 4 provides a perspective on future trends and open research opportunities in this rapidly evolving area.

## Surface Instability Mechanism

**Fig. 2:**
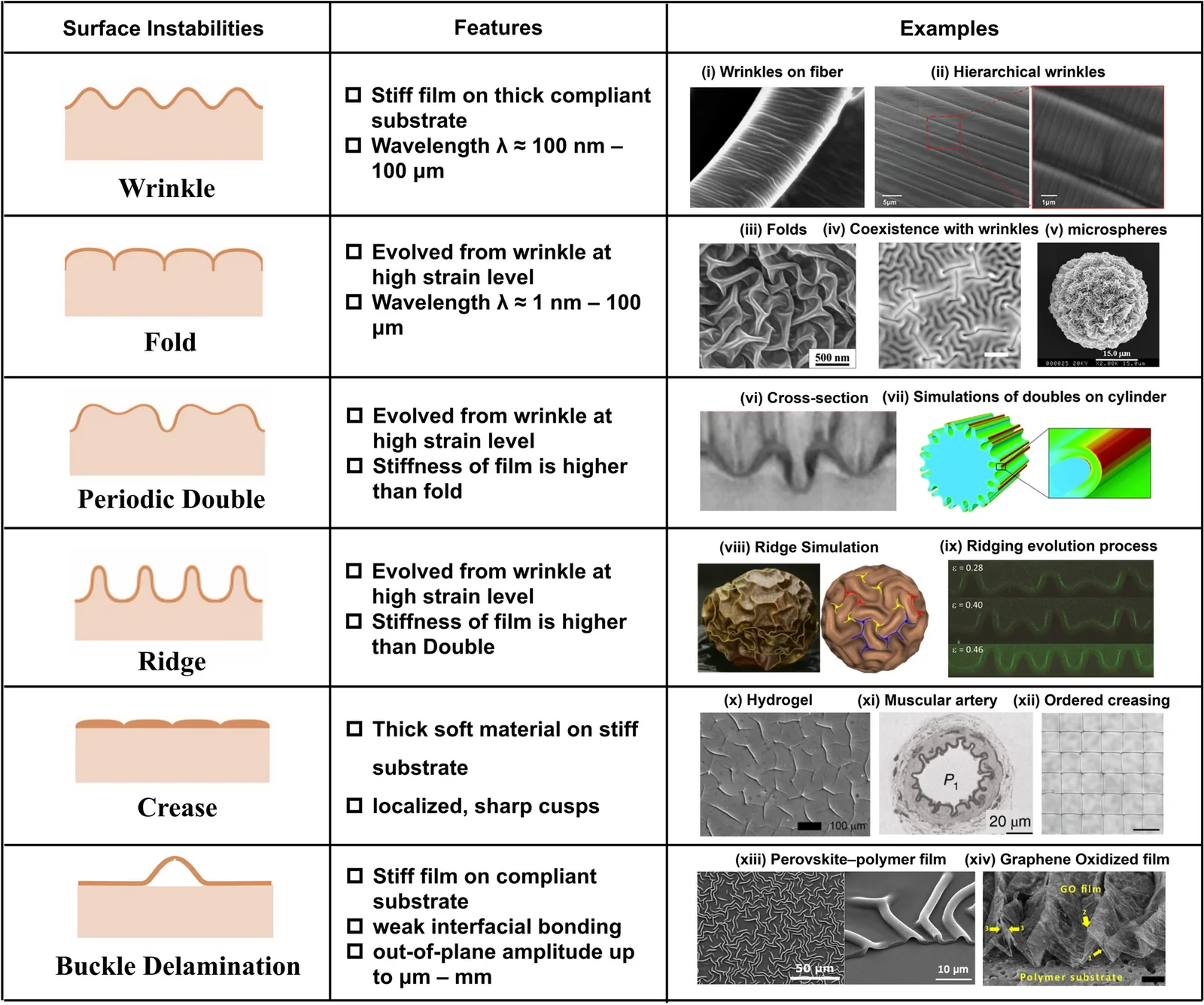
A summary of morphologies, features and examples of surface instabilities: wrinkle, fold, Periodic double, ridge, crease and delamination buckle. Images (i)—(xiv) are reprinted with permission from Refs. [12, 62–74]

The formation of surface instabilities is a complex dynamic evolution process. When the strain mismatch between a film and its substrate exceeds a critical threshold, the resulting compressive stress in the film can render the initially flat surface mechanically unstable, leading to distinct instability modes that minimize the system’s strain energy [48–55]. Typical modes include wrinkles, creases, folds, delaminated buckles, period-doubling patterns, and ridges, with the specific pattern determined by the material properties, interfacial characteristics, geometric dimensions, and the degree of strain mismatch [56–61]. Figure 2 summarizes the fundamental mechanical models governing these surface instabilities in planar systems, along with representative examples from the literatures.

### Strain Mismatch to Generate Surface Instabilities

Within the framework of continuum mechanics, the stress state in a thin film bonded to a substrate can be quantitatively described using the classical Stoney formula, which relates the average in-plane stress in the film to the curvature of the film–substrate system. Specifically, strain mismatch arising from thermal expansion, swelling, or growth-induced effects is translated into macroscopic bending of the substrate due to the constraint imposed by interfacial bonding. This curvature reflects the accumulation of tangential shear stress at the film–substrate interface, which serves as the primary driving force for surface instabilities. When the compressive stress stored in the film exceeds a critical threshold, the system lowers its total elastic energy through out-of-plane deformation, leading to the formation of wrinkles or folds. Although originally derived for a single thin film on a thick substrate, the Stoney formulation provides a first-order estimation of the interfacial stress level and offers a fundamental link between microscopic stress accumulation and the macroscopic manifestation of surface instability, thereby laying the theoretical foundation for predicting instability onset and characteristic microstructural features.

**Fig. 3:**
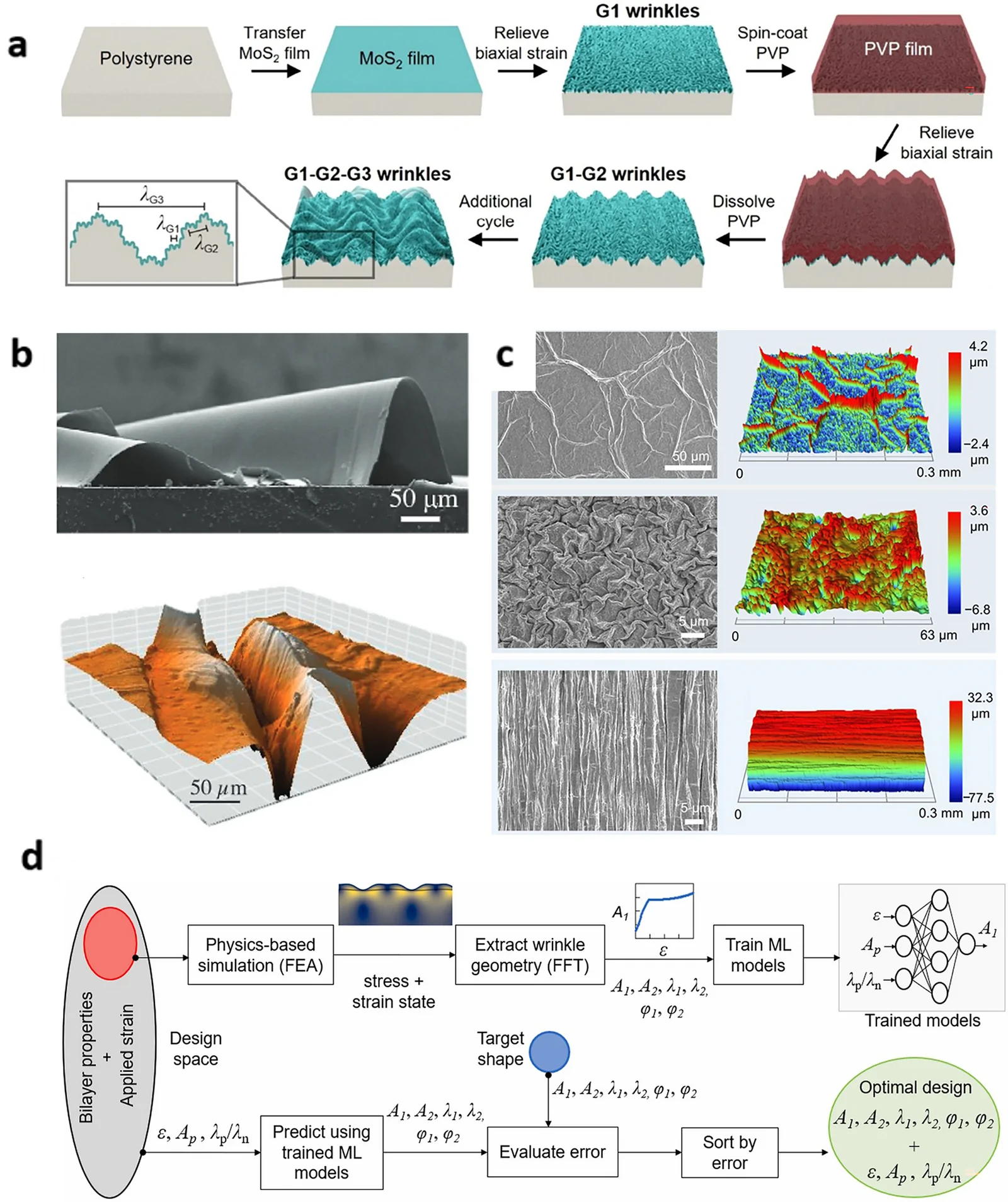
**a** Scheme describing the transfer process and formation of wrinkled MoS_2_. Reproduced from reference [81]. **b** SEM and AFM (3D topography) images of WS_2_ wrinkle structure. Reproduced from reference [82]. **c** Three typical wrinkling modes of graphene oxides characterized by SEM. Reproduced from reference [88]. **d** Overview of the inverse design of hierarchical wrinkling parameters based on machine learning. Reproduced from reference [100]

Strain mismatches in film–substrate systems can be introduced through a variety of physical and chemical mechanisms, each of which generates in-plane compressive or tensile stresses that may trigger surface instabilities. The first strategy is based on mechanical pre‑strain. Its core mechanism involves releasing the stored elastic energy from a pre‑stretched substrate to induce compressive stress in an attached film [34, 75–80]. For example, Rhee et al. present a solution-based hierarchical wrinkling approach to create continuous, stretchable semiconducting MoS_2_ films over wafer-scale areas (Fig. 3a) [81]. Large electrochemically exfoliated MoS_2_ nanosheets are assembled into a uniform film on a prestrained thermoplastic substrate, followed by strain relief to form nanoscale wrinkles. Ghosh et al. generated wrinkled WS_2_ films on PDMS substrates [82]. Chemical vapor deposition (CVD) grown WS_2_ was transferred over a prestrained PDMS substrate. Upon relaxation of the substrate, a large compressive strain occurs in the WS_2_ and induced a buckling delamination phenomenon due to the weak bonding between the WS_2_ film and the PDMS substrate. The morphology characterization using scanning electron microscopy (SEM) and atomic force microscopy (AFM) clearly reveals the wrinkle generation over the entire film with the average height of an individual wrinkle being around 100–120 µm (Fig. 3b).

Swelling- or shrinkage-induced strain mismatch typically originates from osmotic pressure changes such as solvent uptake or release in polymeric films and thermal expansion upon heating or cooling [83–87], where nonuniform volumetric deformation is constrained by the substrate, leading to the accumulation of planar mismatch strain. For example, Li et al. generated wrinkled graphene oxide sheets on drying-induced shrinking hydrogel substrates, at the same time uncovered selection rules determined by curvature mismatch between the sheets and target surfaces [88]. Three wrinkling modes are identified: isotropic cracked land patterns on flat surfaces, labyrinthine patterns on spherical surfaces, and anisotropic curtain phases on cylindrical surfaces (Fig. 3c). Photo-triggered transformations, including photochemical reactions, photoisomerization, or localized photothermal heating, can induce spatially or temporally controlled strain by altering molecular configurations or thermal fields within the film [89–96]. Another strategy utilizes substrate geometry and patterning. The underlying principle relies on guiding and customizing the instability morphology by designing the initial shape or surface pattern of the substrate [97–99].

Although surface instabilities are increasingly exploited as functional microstructures rather than being regarded as mere failures, the formation and evolution of these instabilities are still bounded by intrinsic structural failure limits. As compressive strain continues to increase, classical wrinkling modes may undergo secondary instabilities, such as period-doubling, folding, or ridge localization, which can further trigger material damage including interfacial debonding, crack initiation, or surface crackling. These failure phenomena are often governed by a competition between geometric nonlinearity, material nonlinearity, and interfacial strength. For instance, excessive curvature concentration at wrinkle crests or fold hinges can lead to localized tensile stresses exceeding the fracture strength of stiff films, while accumulated shear stresses at the film–substrate interface may promote delamination. Therefore, understanding the failure thresholds and transition pathways from reversible instability to irreversible damage is essential for the reliable design of mechanically programmed microstructures. Recent studies have begun to establish failure maps linking instability modes with fracture or delamination criteria, providing important guidelines for extending functionality while avoiding catastrophic microstructural failure. For example, Zhang et al. found that both globally periodic buckle-delaminated patterns and ordered cracking patterns over large areas could be observed in spontaneously buckle-delaminated metal or silicon films [101]. By patterning the films into ribbons with widths smaller than the predicted cracking periodicity, they successfully generated crack-free spontaneous delaminated ribbons on highly prestrained elastomer substrates.

Overall, researchers have developed a variety of methods for processing materials to engineer surface instabilities. These mechanisms offer versatile pathways to tailor the magnitude, distribution, and reversibility of in-plane strain, thereby enabling precise control over the onset and evolution of wrinkling and folding microstructures.

### Predict Surface Instability Morphologies via Mechanical Modeling

The ability to precisely control morphology formation is essential for the rational design of multifunctional surfaces with tailored properties. The diversity of surface instability phenomena arises from the nonlinear post-buckling mechanics of thin film–substrate systems. The film and substrate interact in a highly coupled, energy-driven process where multiple deformation pathways compete. The system naturally evolves toward a configuration that minimizes total energy under the given constraint, which includes bending energy of the film, stretching energy of both film and substrate, interfacial energy from adhesion or delamination, and external work from driving force [102–111]. Neglecting nanoscale surface effects, thin films can be treated as continuum elastic plates, and the energy minimization approach is commonly employed to analyze the nonlinear surface instabilities on bilayer film–substrate systems [112–120].

#### Wrinkling

A widely adopted theoretical framework models the system as a thin, stiff film bonded to a compliant substrate, providing a classical basis for analyzing wrinkling behaviors. Uniaxial compression readily produces periodic stripe-like wrinkles with controllable wavelength and amplitude across large areas, which can be achieved by releasing prestretched film–substrate assemblies [75]. While biaxial compression generates more diverse surface morphologies, ranging from randomly distributed labyrinth patterns [78] to highly ordered hexagonal or herringbone configurations. These morphologies can be further controlled by modulating the stress release sequence and the biaxial stress state. Yin et al. demonstrated a design guideline for tuning two-dimensional herringbone patterns via sequential wrinkling, enabling quantitative control over complex wrinkle architectures [121]. If the prestretch strain is small (less than 5%), small deformation model predicts the wrinkling wavelength and amplitude of sinusoidal wrinkles as [112]:

1 $$\lambda = 2\pi t_{f} \left( {\frac{{\overline{E}_{f} }}{{3\overline{E}_{{{\mathrm{sub}}}} }}} \right)^{\frac{1}{3}}$$

2 $${\mathrm{A}} = \frac{\lambda }{\pi }\sqrt {\left( {\varepsilon - \varepsilon_{{{\mathrm{cr}}}} } \right)}$$

where $\overline{E }=\frac{E}{1-{\nu }^{2}}$ is the plane-strain modulus, *E* is Young’s modulus and ν is Poisson’s ratio. The subscripts “*f*” and “sub” stand for the film and substrate, respectively. ${\varepsilon }_{cr}$ is defined as the critical buckling strain. Equations (1) and (2) suggest that both the wavelength and amplitude are linearly proportional to the film thickness and only the amplitude increases with the prestretch strain quadratically. That means, due to the assumption of small strain, the prestretch strain is released through the increase of the buckling amplitude while the wavelength remains unchanged. For finite deformation (large than 5%) model, the finite deformation and geometrical nonlinearity of soft substrate must be taken into account, and the wrinkling wavelength and amplitude could be related to those of the small strain case as [122]:

3 $$\lambda = \frac{{\lambda_{0} }}{{\left( {1 + \varepsilon } \right)\left( {1 + \xi } \right)^{\frac{1}{3}} }}$$

4 $$A = \frac{{A_{0} }}{{\left( {1 + \varepsilon } \right)\left( {1 + \xi } \right)^{\frac{1}{3}} }}$$

Here ${\lambda }_{0}$ and ${A}_{0}$ are the wrinkling wavelength and amplitude for the small strain case given by Eqs. (1) and (2). ${\left(1+\xi \right)}^\frac{1}{3}$ comes from the nonlinearity of finite deformation with ξ =$\frac{5\varepsilon \left(1+\varepsilon \right)}{32}$.

Beyond planar substrates, growing attention has been directed toward exploiting surface instabilities on curved geometries, inspired by the wrinkling morphologies widely observed in biological systems. Curved substrates such as cylinders, cones, spheres, and tubular structures offer unique platforms for mimicking biological wrinkling processes and advancing bioinspired engineering [123]. In living organisms, surface wrinkles and folds typically arise from mechanically driven instabilities induced by growth, differential stiffness, or internal stress mismatches between layered tissues. For instance, compressive stresses generated during tissue growth or hydration mismatch between epithelial layers and underlying substrates can trigger buckling and wrinkling phenomena, as widely observed in skin, gastrointestinal mucosa, and brain cortices [124, 125]. These naturally occurring wrinkled morphologies are not merely structural byproducts, but play essential biological roles, including enhancing surface area for mass transport, regulating cell migration and differentiation, accommodating large deformations, and improving mechanical compliance and frictional performance [126].

From a mechanistic perspective, numerous studies have demonstrated that the formation of biological wrinkles can be effectively interpreted within the same framework of elastic instability and energy minimization principles that govern artificial wrinkling systems, despite additional biological complexities such as active cellular forces and biochemical remodeling [127]. This consistency has motivated extensive biomimetic efforts aimed at reproducing biological wrinkling functions on engineered curved substrates, enabling controllable hierarchical morphologies for applications in tissue engineering, artificial organs, and functional soft interfaces [40, 128]. A representative example was reported by Chan et al. [129], who demonstrated a biomimetic approach by generating wrinkled patterns on cell-laden hydrogel films bonded to prestretched hydrogel substrates. This system recapitulated mucosal folding processes and provided a proof-of-concept platform for programmable self-folding artificial mucosa. Such biofabrication strategies not only offer deeper insights into the biomechanical, biochemical, and physiological properties of biological wrinkled interfaces, but also open new opportunities for biomedical applications in artificial organ development, drug delivery systems, and soft robotic devices.

#### Folding, Ridging, and Periodic Doubling

Folding, ridging, and period-doubling phenomena can be regarded as advanced evolutionary modes of wrinkle patterns under high compressive strain. As the applied compressive strain increases beyond the primary wrinkling instability, the initially sinusoidal wrinkle morphology progressively undergoes nonlinear amplification, symmetry breaking, and mode interaction, giving rise to secondary instabilities. These secondary instabilities manifest as localized folding, ridge formation, or periodic doubling of the wrinkle wavelength, reflecting a transition from linear to strongly nonlinear deformation regimes. Such hierarchical evolution of surface morphologies is governed by the interplay among material nonlinearity, geometric confinement, and interfacial constraints, and provides a versatile pathway for achieving complex and programmable surface structures.

Folding is a localized bending which normally originates from the later stage of wrinkling on an elastic substrate [130–132]. The amplitude of folds is linearly proportional to the displacement which is in sharp contrast with the wrinkling amplitude that depends on the applied strain. From the scaling law, the radius of fold tip, i.e., the position of the maximum curvature, could be approximated given by [133]:

5 $$R_{f} \sim \left( {\frac{B}{{K{\Delta }^{2} }}} \right)^{\frac{1}{2}}$$

where *B* and *K* are defined in bending stiffness of the film and the substrate, respectively.Δ is the film displacement with respect to the compression. Brau et al. suggest that the fold forms progressively starting with multiplication of wrinkling wavelength and ending with self-contact [134].

#### Creasing

Creasing represents a distinct instability mode that typically occurs in incompressible soft materials bonded to rigid substrates. Creasing emerges through a nucleation and subsequent growth process, driven by the energetic competition between bulk elastic deformation and surface energy. Upon sufficient compressive loading, the free surface undergoes localized self-contact to minimize elastic energy, resulting in sharply indented slit-like or tri-lobed creases [135, 136]. Unlike wrinkles, which initiate at small strains, creases emerge at higher strains and can be triggered or accelerated by surface imperfections or defects [137–139]. The energy difference between the flat and creased configurations is given by the following expression [140]:

6 $$\Delta U = U_{{{\mathrm{crease}}}} - U_{{{\mathrm{flat}}}}$$

where *A*, *B*, and *C* are positive constants, γ is surface tension, μ is shear modulus, *a* is length of a self-contact region, ε is the compressive strain, and ε_0_ is the critical strain without surface tension effects. The dominant contributions are the first two terms on the right-hand side of the equation. The first term arises from surface energy and acts to suppress crease nucleation, whereas the second term is associated with elastic energy and favors the formation of a creased state. Consequently, the system must overcome an energy barrier to initiate a crease, which requires a sufficiently large compressive strain. There is also the natural length of a crease spacing [140]:

7 $$\lambda_{{{\mathrm{crease}}}} = \, 3.5H(1 \, - \varepsilon )$$

However, creased surfaces exhibit much less spatial order than wrinkled surfaces. Unlike wrinkling, which selects a characteristic wavelength through a bifurcation-driven instability, creasing is a localized, nonlinear nucleation process that is strongly influenced by imperfections and stress fluctuations, leading to irregular spacing and poor periodicity.

#### Buckle Delamination

When the bonding between a film and its substrate is not strong enough to withstand the tension stress, the film may detach from the substrate. The delamination typically appears as localized blisters either strip-like [97] or circular shape [141] depending on stress state. Vella et al. have developed the scaling laws for the critical size ${l}_{d}$ of the blister which defined as the initial length of strip-like blister or the initial diameter of circular blister. For a wide strip on a thick substrate, ${l}_{d}$ scales as

8 $$l_{d} \sim \left( {\frac{{B^{2} }}{{E_{{{\mathrm{sub}}}} \Gamma }}} \right)^{\frac{1}{5}}$$

Based on Griffith’s criterion, Γ equals to the work of separating an interface. When the stress is beyond the critical threshold, the cracks surrounding the blister grow further. Hutchinson et al. show that in the initial stage of the crack growth, the blister remains nearly circular [141]. As the stress further increases, the crack growth becomes unstable and large area of delamination occurs. Zhang et al. studied the buckling induced periodic delamination of the stiffer film on hyperelastic substrate under extremely large compressive strain [101, 142, 143]. The shape evolution of the buckled film and theoretical predictions of the size of blisters were given. Lin et al. investigated the delamination of a thin patch attached on a dynamic wrinkling surface [144]. The analytical expression for the critical delamination strain and strain energy release rate was derived. The equilibrium blister size after delamination is given by:

9 $$\delta = \frac{{\pi h_{p} \sqrt {\overline{E}_{p} h_{p} \varepsilon } }}{{\sqrt {\Gamma - U_{{{\mathrm{diff}}}} } }}$$

where ${\overline{E} }_{p}$ and *h*_*p*_ is the plane-strain modulus and the thickness of the thin patch, respectively. And *U*_diff_ is the total strain energy density difference of the whole system between the wrinkling of composite bilayer on substrate and the wrinkling of only thin patch on substrate. A smaller *U*_diff_ is beneficial for retarding the growth of delamination.

#### Surface Instability with Hierarchical Structures

Beyond simple periodic wrinkles, hierarchical wrinkling structures with spatially varying wavelengths offer additional opportunities to program both global and local surface functionalities, thereby expanding the range of potential applications [145–147]. Compared with single-level wrinkles, hierarchical wrinkles demonstrate a higher capacity for strain accumulation, which benefits the performance of stretchable devices. They also enable multiple modes of morphological transition, offering greater flexibility in tuning surface properties. The formation mechanisms of hierarchical wrinkling can be broadly classified into two categories: (i) subsequent local wrinkling occurring on an initially wrinkled surface, and (ii) subsequent global wrinkling of the initial wrinkled surface. When the film thickness is relatively small, local wrinkling is favored. The resulting wrinkles have a characteristic size smaller than the wavelength of the initial sinusoidal pattern. This phenomenon, along with its underlying mechanics, has been well explained using a cylindrical core–shell model [148]. As the film thickness increases beyond a critical value, the overall bending stiffness of the corrugated coating governs the buckling behavior, leading to global wrinkling as revealed by Lin and co-authors [63]. In this case, the corrugated coatings deform in a manner similar to flat films during buckling, producing wrinkles with wavelengths larger than those of the initial sinusoidal corrugations. Consequently, hierarchical surface patterns formed via global wrinkling appear as an organized, self-similar structure—small wrinkles superimposed upon larger ones.

Over the past decade, the design paradigm for microstructures has undergone a revolution, driven primarily by the deep integration of artificial intelligence (AI) and inverse design principles. By leveraging FEA to accurately simulate the mechanical processes of surface instabilities and functional responses, a “design–performance” dataset is generated. Machine learning is then used to construct an inverse mapping from the target performance to design parameters (such as film thickness distribution, modulus gradient, or prestrain field). For example, Saha utilized nonlinear FEA to simulate the compressive instability process of prepatterned bilayer films [100]. By systematically varying the prepattern period (λₚ), amplitude (Aₚ), and compressive strain (ε), a dataset of “input parameters–wrinkle geometry parameters” was generated. Five independent shallow neural networks were trained to predict the two wrinkle amplitudes (A₁, A₂), the period of the second mode (λ₂), and the two phase angles (φ₁, φ₂) (Fig. 2d). This model can rapidly identify the optimal combination of process parameters by minimizing the error between the predicted wrinkle profile and the target profile, enabling precise control over complex microstructures such as flat-topped sinusoidal waves.

#### Synthesis and Summary

Although the governing equations introduced above appear distinct in form, the various surface instability modes share a common physical origin rooted in energy minimization under mechanical constraint. In all cases, instabilities arise from the competition between destabilizing compressive strain and stabilizing energetic contributions, including film bending stiffness, substrate elasticity, surface energy, and interfacial adhesion. Wrinkling represents a primary bifurcation instability, where a characteristic wavelength is uniquely selected through the balance between bending and elastic energies, leading to spatially periodic and ordered patterns. As the applied strain increases, secondary instabilities such as folding and period-doubling emerge due to geometric and material nonlinearities, reflecting a progressive departure from the linear stability regime. In contrast, creasing is a strongly nonlinear, localized instability governed by a nucleation and growth mechanism, in which elastic energy gain competes with surface energy penalty, resulting in an activation energy barrier and the absence of a well-defined intrinsic wavelength. Delamination-induced buckling further incorporates interfacial fracture energy into this framework, coupling mechanical instability with damage evolution. From this unified perspective, the different analytical expressions in Sect. 2.2 can be viewed as mode-specific manifestations of a common energetic framework, with the observed morphological diversity arising from differences in dominant energy terms, nonlinearity, and interfacial constraints.

## Applications of Surface Instabilities

### Electronic Skin (E-skin)

Electronic skins (E-skins) are designed to emulate the sophisticated human somatosensory system by transducing physical signals such as pressure, humidity, and temperature into electrical signals, with potential applications in robotics, human–machine interfaces, and health monitoring [149–151]. In many E-skin architectures, surface instability-induced microstructures naturally arise from elastic modulus mismatch, prestrain release, or stress redistribution between thin films and soft substrates. Specifically, surface instabilities can increase the effective contact area, enable large and reversible deformation without mechanical failure, and modulate local stress and strain distribution, thereby enhancing sensitivity, stretchability, durability, and signal stability of E-skin devices. As a result, surface instability engineering has become an effective and scalable strategy for improving E-skin performance beyond what is achievable with planar device configurations.

#### Enhanced Sensing Capacity for E-Skin

Pressure sensors based on piezoresistivity, capacitance, or piezoelectricity have demonstrated high sensitivity and excellent flexibility. Microstructured surfaces have been fabricated to further enhance sensitivity and adjust the operating range [98, 152–156]. In particularly, surface wrinkling structures flatten under out-of-plane compression, and this change in wrinkling amplitude in response to external pressure has been utilized in the fabrication of pressure sensors [157–161].

Zeng et al. reported a tunable, ultrasensitive (14. 268 kPa^−1^) and flexible capacitive pressure sensor with a low detectable pressure limit (1.5 Pa) [162], a fast response time (< 50 ms), and a cycling stability over 1000 cycles at 0.15 kPa, based on a wrinkled PDMS microstructures. The wrinkled microstructure is replicated from a reusable PDMS mold fabricated by releasing a prestretched PDMS film treated with UVO. The sensitivity and working range of the sensor could be modulated independently by changing the morphology of wrinkles in a well-controlled manner. The authors demonstrated the capability of monitoring the respiratory rate and recognizing different words using this sensor, showing its promising application potential for disease diagnosis and prosthetics.

In a similar vein, Yang et al. introduced a flexible capacitive pressure sensor that integrates thermal stress-induced microstructured ionic gels with textile electrodes in a band-aid-like form [163]. This design simplifies production by eliminating the need for external templates or complex fabrication processes while enhancing performance. The ionic gel forms an efficient electric double layer (EDL) that converts ion–electron interactions into measurable pressure responses. In this system, the presence of surface instability-induced microstructures plays a critical role by increasing and dynamically modulating the effective interfacial contact area. Upon applied pressure, deformation and partial flattening of the microstructured ionic gel significantly enlarge the EDL-active area, leading to a pronounced capacitance change. This microstructure-enabled amplification mechanism underlies the high sensitivity and wide working range of the sensor. As a result, the sensor offers impressive sensitivity (5652.41 kPa⁻^1^), a broad pressure range (0–400 kPa), rapid response, excellent long-term stability (over 1000 cycles), and a low detection threshold.

**Fig. 4:**
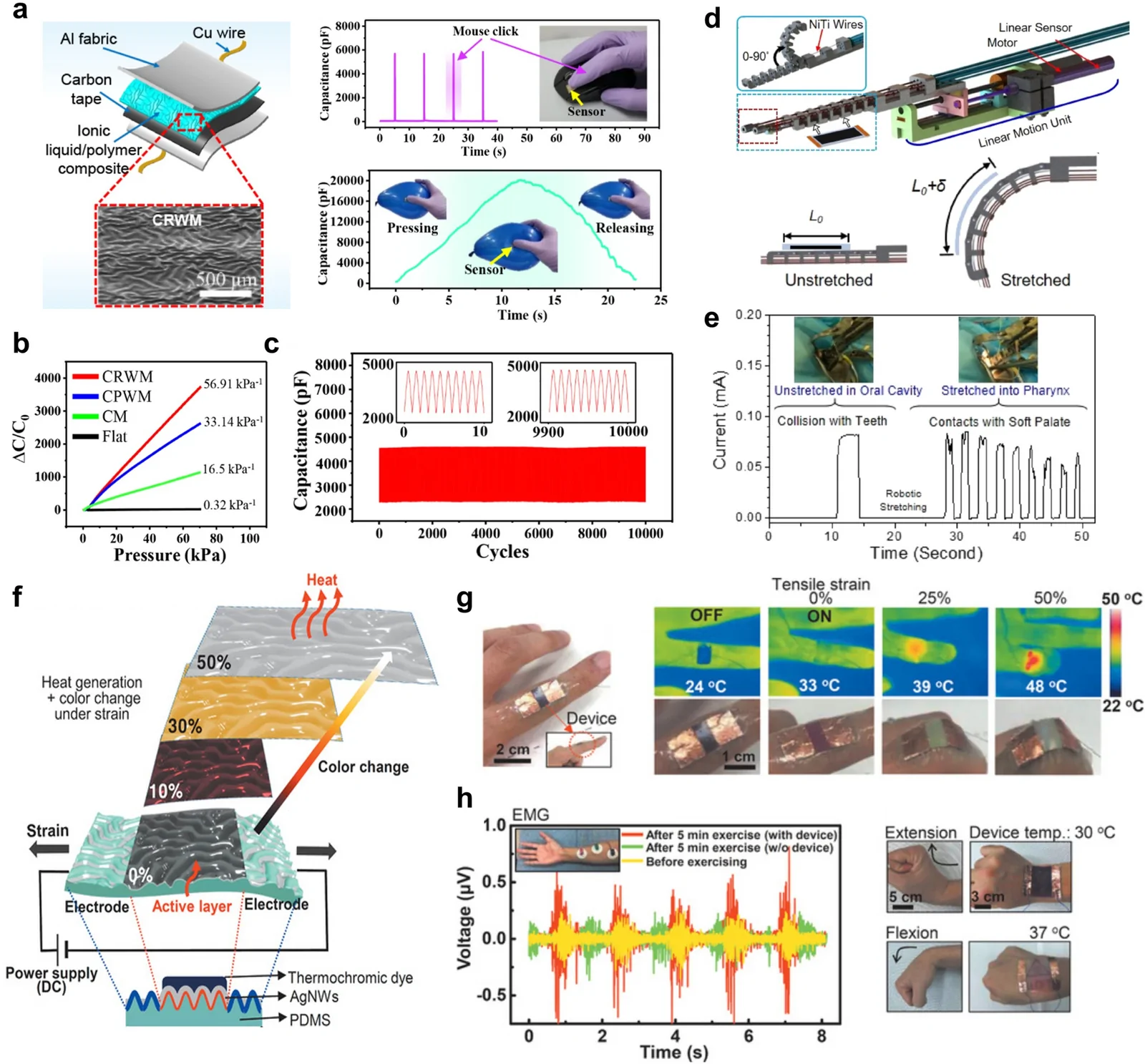
**a** Structure and E-skin application of the wrinkled IL/polymer composite pressure sensor. **b** Sensitivity of the wrinkled IL/polymer composite pressure sensor. **c** Cycling test of wrinkled pressure sensor showing stable signal for 10,000 cycles. Reproduced from reference [164]. **d** Top: Surgical robots with the wrinkled rGO pressure sensor for the collision-aware. Bottom: The stretching actuation of the rGO pressure sensor and manipulators. **e** Real-time monitoring of the robot − tissue collision/interaction during the cadaveric testing using the collision-aware surgical robots with wrinkled rGO pressure sensor working at unstretched and stretched states. Reproduced from reference [165]. **f** Schematic diagram and working mechanism of the integrated stretchable device inducing heat generation and color change under tensile strain for thermotherapeutic rehabilitation. **g** Heat and color changes of the stretchable device observed with IR and optical cameras on human finger. **h** Left: EMG signals during finger motions with and without device before and after exercise. Inset: positions of the EMG detection electrodes on the forearm. Right: Large‐scale application of the device to joint movement (extension and flexion) of the wrist. Reproduced from reference [168]

To further advance the durability and sensitivity of E-skin, Cho et al. fabricated a capacitive pressure sensor using an ionic liquid (IL)/polymer composite with a randomly wrinkled microstructure (Fig. 4a) [164]. Four types of microstructures—convex and randomly wrinkled microstructures (CRWM), convex and parallelly wrinkled microstructures (CPWM), convex but smooth microstructures (CM), and flat microstructure—were examined and compared to reveal the effect of microstructures on the sensor performance. The wrinkled sensor achieved the highest sensitivity of 56.91 kPa^–1^ and linearity over a wide range (0–80 kPa) and could detect a pressure of as small as 0.5 Pa (Fig. 4b). This sensor further showed a stable signal during over 10,000 loading–unloading cycles (Fig. 4c) showing significantly improved durability. The sensor was demonstrated as a potential E-skin for robotics. It attached to a glove fingertip to detect the user’s finger tapping, and it was manufactured as a sensor array to measure the weight of objects and predict their shape.

Collision-aware surgical robotics could improve the safety and efficiency of minimally invasive surgery in a confined space. Chang et al. developed a stretchable pressure sensor consisting of reduced graphene oxide (rGO) electrodes with biomimetic topographies inspired by the multidimensional wrinkles of Shar-Pei dog’s skin [165]. The wrinkle-crumple rGO electrodes exhibiting high stretchability (~ 100%) and strain-insensitive resistance profiles (a gauge factor < 0.05) were utilized to fabricate piezoresistive pressure sensors. The stretchable pressure sensors were integrated with two surgical robots to detect the robot-tissue contacts in real time for the transoral robotic surgery procedure (Fig. 4d, e). Similarly, Jia et al. also developed the hierarchical wrinkled rGO-based pressure sensor [166]. Benefiting from the skin-like wrinkling structure, the pressure sensor demonstrates outstanding sensitivity, reaching 178 kPa^−1^. It can also detect pressure as small as 42 Pa. Furthermore, continuous gradients were introduced into the wrinkles to prepare a gradient wrinkle sensor that realizes the position and motion detection of moving objects. Tang et al. presented a pressure sensor made of reduced graphene oxide (rGO) and cellulose nanocrystals (CNC) that offers rapid response, high stability, and durability, making it ideal for E-skin and wearable sensor applications [167].

Interference between sensing components and the difficulty in synchronous monitoring are practically encountered when electronic skins are applied to mixed signals. User-interactive electronic skin with a distinguishable output is promising for human–machine interfaces and healthcare applications. Lee et al. reported a user-interactive thermotherapeutic electronic skin which is fabricated by combining thermochromic composites and stretchable strain sensors [168]. The strain sensors were fabricated using strain-responsive silver nanowire networks on microwrinkles. Synchronous color and heat of the electronic skin could be easily controlled through electrical resistance variation induced by applied mechanical strain (Fig. 4f). The application of thermotherapy on human fingers (Fig. 4g) and the monitoring of EMG signals (Fig. 4h) were demonstrated.

#### Integrated with TENG for Energy Harvesting and Self-Powering

The triboelectric nanogenerators (TENG) are considered to be a promising method for energy harvesting from environment [169, 170]. The large specific area provided by the wrinkling surface structure has been proven as an effective way to increase the performance of the TENG [171]. Most recently, great effort has been put by researchers to integrate the triboelectric nanogenerator and the sensors together for self-powered E-skins [133, 172, 173]. Amazingly, it was found the surface wrinkling could enhance both the performance of the triboelectric nanogenerators and sensors in terms of flexibility, efficiency and sensitivity.

Cho et al. introduced a hierarchical wrinkled-TENG (HWA-TENG) with dual-wavelength structures (3.1 µm microscale and 311.8 nm nanoscale) [79], characterized by its deformability, biocompatibility, ultrathin profile, and flexibility. Utilizing a plasma-polymer-fluorocarbon (PPFC) thin film as the triboelectric material, the device achieves a high surface charge potential of 7.28, comparable to bulk polytetrafluoroethylene. The wrinkled architecture increases the surface area by 3.5%, resulting in a high output performance of 200 V and 30 µA. With its eco-friendly fabrication process and high efficiency, the HWA-TENG demonstrates broad applications, including triboelectric raindrop energy harvesting and wearable, conformal triboelectric devices for human use. Gu et al. utilized a piezopotential-enhanced triboelectric effect through a hierarchical wrinkle structure of PDMS/ZnO NWs [174], thereby developing a triboelectric pressure sensor (TPS) (PETPS) with high sensitivity and a broad detection range. The deformation of ZnO nanowires enhances charge transfer, while the hierarchical structure enables a self-adjustable contact area. This design achieves high sensitivity (0.26 nC cm⁻^2^ kPa⁻^1^ from 1 to 25 kPa and 0.02 nC cm⁻^2^ kPa⁻^1^ from 25 to 476 kPa), fast response time (46 ms), a wide sensing range (1 to 476 kPa), and excellent stability (over 4000 cycles).

Inspired by the human fingerprint, Kang et al. developed a pressure sensor with energy-harvesting functions based on the conducting hierarchical wrinkles [79], which composed of PDMS wrinkles as the primary microstructure and embedded Ag nanowires as the secondary nanostructure. The hierarchical wrinkle-based conductor could harvest mechanical energy via contact electrification and electrostatic induction and deliver an average output power of 3.5 mW with an open-circuit voltage of 300 V and a short-circuit current of 35 µA. The hierarchical wrinkle-based conductor was also used as self-powered tactile pressure sensor with a sensitivity of 1.187 mV Pa^−1^ in both contact-separation mode and the single-electrode mode.

### Stretchable Devices

The past decade has witnessed significant growth in the field of stretchable devices [175, 176]. The electronics with soft and stretchable form factors can operate stably in dynamic in vivo environments subjected to repetitive movements, achieving seamless integration with biological tissues due to their exceptional mechanical compliance. For example, Chang et al. discussed the development of fiber-based electrochemical sweat sensors that enable real-time health monitoring by detecting biomarkers in sweat, providing personalized health feedback [149]. Wu et al. introduced liquid metal core-sheath fibers, which are highly stretchable and maintain stable resistance, making them suitable for ultrasensitive physiological monitoring [177]. Xu et al. further explored the use of smart textiles embedded with sensors for monitoring physiological conditions, highlighting their promising applications in personalized sports tracking and healthcare [42]. This section focuses on stretchable electronic components beyond sensing functions, including stretchable batteries, displays, and other active or energy-related devices, which serve as fundamental building blocks for mechanically compliant electronic systems.

New generation stretchable devices are designed to provide even more advanced functionalities [178, 179]. However, the realization of such complex designs is usually hindered by the flexibility of devices. To face this emerging field, it is critical to develop novel stretchable structures in electronics to accommodate deformation and maintain stabilization. One of the major strategies to achieve flexibility and stretchability is to utilize surface instability to generate wavy structures [180]. Wrinkling-based fabrication methods, such as prestrain release and thin film buckling, have been adopted to create flexible and deformable electronic architectures. These designs maintain excellent electrical and optical properties even under large mechanical strain, with certain stretchable LEDs exhibiting more than 70% performance improvement compared with their planar counterparts [181, 182]. Control over wrinkle hierarchy and microscale geometry further enhances mechanical robustness, stretchability, and luminance stability during repeated deformation, supporting the development of wearable integrated electronic systems [183].

#### Stretchable Batteries

Batteries are typically composed of five components: anode, cathode, electrolyte, separator, and current collector, all of which are rigid and thus, cannot resist nearly any strain [184]. The design of flexible batteries has been extensively studied. Among all previous efforts, one promising direction is to utilize the surface instabilities to achieve high stretchability in batteries. The formation of wavy structure enables the rigid components of batteries to accommodate large deformation [181].

Several studies have reported a component-level strategy to achieve stretchability of each individual component in batteries which can be assembled to develop a stretchable battery. For example, the wavy designs were exploited in the creation of stretchable electrodes made of graphenes [99, 185, 186], carbon nanotube (CNT) films [187], and organic polymers [188]. Jeong et al. controlled the wrinkle textures of a free-standing graphene nanosheet (GNS) for the design of stretchable graphene-based battery electrodes [189]. These wrinkles enhanced Li-ion diffusion into the voids and increased the surface area. Consequently, GNS-based electrodes exhibited a high specific capacity of ~ 740 mAh g^−1^ at 100 mA g^−1^ and the greater power capability with ~ 404 mAh g^−1^ being delivered even at 2 A g^−1^. Chen et al. also showed similar results that the resulting graphene electrode had large reversible stretchability [190], high specific capacity, high-rate capability, and long-term cycling stability.

In addition, organic polymer also can be utilized as buckled, stretchable electrodes for battery applications. Wang et al. developed 2D buckled polypyrrole (PPy-*p*TS) biofilms on prestrained gold coated elastic substrate as stretchable electrode [188]. This electrode remained high performance after 2000 stretching cycles with 30% applied strain in Mg batteries. Cao et al. created a fully stretchable solid-state lithium-ion battery (FSSLIB) by integrating stretchable components—current collector, anode/cathode, and electrolyte—using crumpled nanowires (NWs) and cross-linked hydrogels [191]. The combination of wrinkled NWs and robust hydrogel electrolytes ensures electrochemical stability during stretching. The FSSLIB achieves a 100% stretch with a specific capacity of 119 mAh g⁻^1^ and maintains 91.6% of its capacity after 250 tensile strain cycles.

**Fig. 5:**
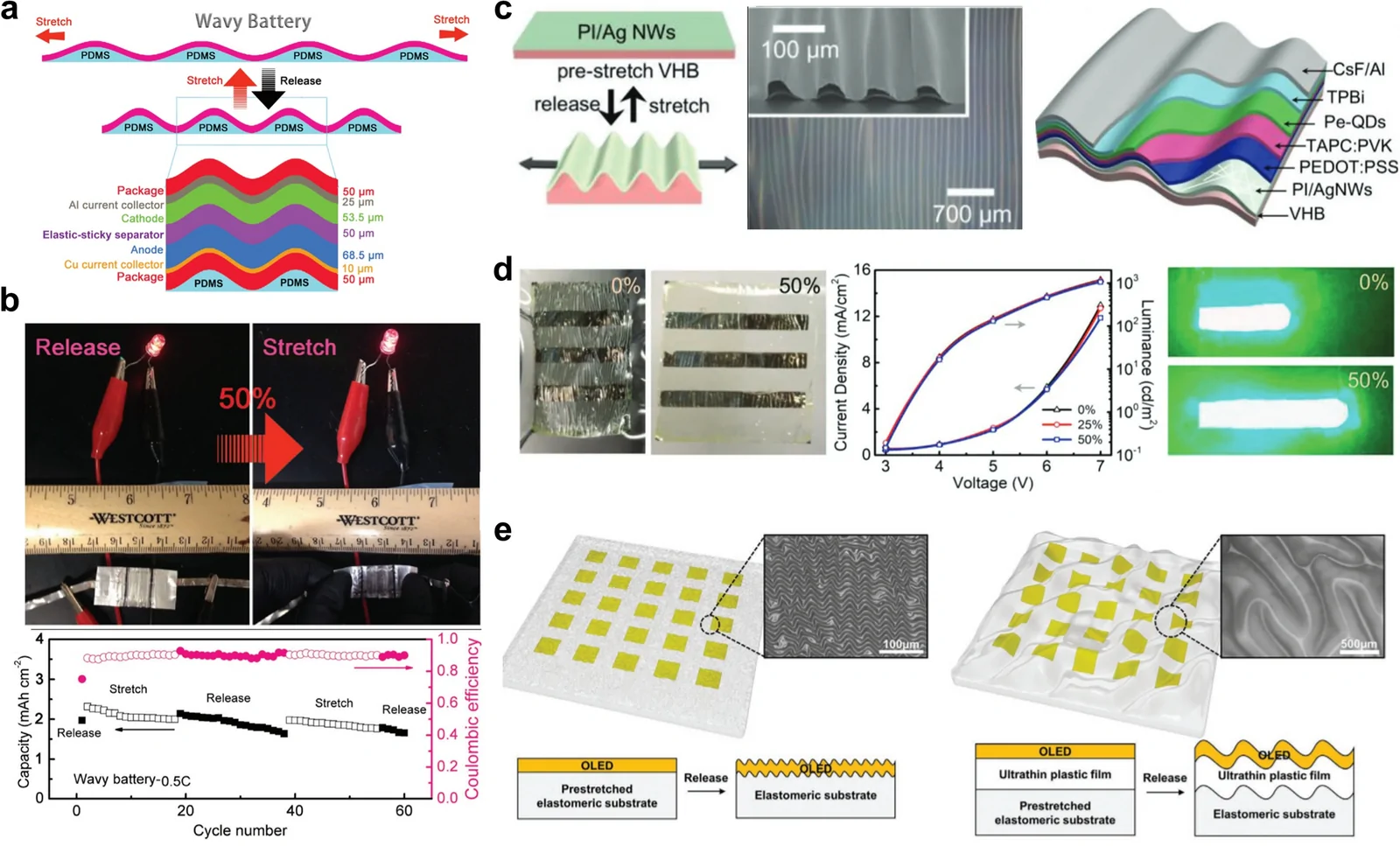
**a** Schematic illustration for the device-scaled wavy battery that all components including cathode and anode and package are stretchable. **b** Cycling performance and Coulombic efficiency for the wavy battery under releasing and stretching states (50% strain). Reproduced from reference [182]. **c** Compositional structure of the perovskite quantum-dot light-emitting diode. **d** Left: Optical images of the Pe-QDLEDs with 0% and 50% tensile strains. Middle: Current efficiency characteristics of stretchable Pe-QDLEDs at specified strain. Right: Optical photographs of the lit stretchable Pe-QDLEDs with strains of 0% and 50%. Reproduced from reference [193]. **e** Schematic illustration of the formation of imperceptible/macroscopic wrinkles in stretchable OLEDs. Reproduced from reference [194]

Similar to the component level, the surface instability method also allows batteries to be stretchable as entire devices, which requires flexibility of each component in battery. Cui and coworkers illustrated a device-scaled strategy to form wavy shape of entire lithium-ion batteries, where all the components can be stretched equally [192]. They pressed the elastic separator into a wavy shape and sealed the crevices of the waves with PDMS (Fig. 5a). With 50% applied strain, this stretchable battery can achieve a high energy density of 172 Wh L^−1^ and areal capacity of up to 3.6 mAh cm^−2^ (Fig. 5b).

#### Stretchable Displays

A critical component of many wearable devices is the stretchable light-emitting diodes (LED) display. However, the commercial applications of stretchable LEDs are largely inhibited by the poor mechanical stability. Attempts have been made to replace active layers in LED with intrinsically stretchable materials. But the device performances are proved not comparable to conventional LEDs [195].

The recent development on surface instability provided another structural approach by utilizing the wrinkle structure. Li et al. fabricated a stretchable organometal halide-perovskite quantum-dot LEDs by employing the LED structure conformed on a surface-wrinkled elastomer substrate [193]. The luminescent efficiency of the device is up to 9.2 cd A^−1^ which is 70% higher than a control diode. The fabrication process of wrinkle structure is by adhering an ultrathin OLED with the thickness less than 3 µm onto a wrinkled PI/AgNWs/VHB composite substrate (Fig. 5c). The resulting wrinkles have a wavelength of ~ 100 µm. With the existence of the wrinkling structure, the device could survive 1000 cycles of stretching with 20% strain and mechanical stretching up to 50% tensile strain will not induce significant loss of the electroluminescent property (Fig. 5d).

One major drawback of the wrinkle-based stretchable LEDs is that the large-sized wrinkles with a few hundred micrometer wavelengths can cause the distortion in the shape of the pixel. To overcome this issue, Jeong et al. directly deposited OLEDs on biaxially prestretched PDMS substrate without the aid of other supporting films via a low-temperature-based solution process and achieved an imperceptible microwrinkles having a wavelength less than 20 µm (Fig. 5e) [194]. The total thickness of the device significantly reduced to 350 nm. The microwrinkled OLEDs show a luminance over 8000 cd m^−2^ and maximum current efficiency of 7.76 cd A^−1^, which is comparable to the device without wrinkled structure. Similarly, in order to improve the display quality of wrinkled OLEDs, Chen et al. developed a simple transfer-free technique to introduce the orderly wrinkles with a period of 79 µm into the stretchable OLEDs [196]. The electroluminescence performance of the stretchable OLEDs with 20% stretchability is comparable to that of the rigid OLEDs on glass substrates. The stretchable OLED based on the wrinkles shows great mechanical stability that the luminance of the device remained at 90% of its initial value after 2000 cycles of stretching.

#### Stretchable Transistors

Stretchable transistors constitute a fundamental building block for next-generation soft electronics, enabling active signal processing under large, reversible mechanical deformations. Advances in stretchable transistor technology enable distributed intelligence directly embedded in deformable substrates, reducing reliance on rigid islands and external signal processing units. This paradigm shift opens new opportunities for adaptive healthcare monitoring, soft robotics, and biointegrated electronics, where real-time decision-making must be maintained under complex, dynamic deformations.

Kim et al. presented a strategy for fabricating stretchable thin film transistors (2D S-TFTs) based on wrinkled heterostructures on elastomer substrates [80], where 2D materials form the gate, source, drain, and channel. The 2D S-TFTs exhibited an initial mobility of 4.9 ± 0.7 cm^2^ V^−1^ s^−1^). Wrinkling reduced strain transfer to the 2D materials by a factor of 50, allowing the substrate to stretch up to 23% with no electrical degradation over thousands of cycles. While stretch did not affect mobility, it induced threshold voltage shifts (ΔV = − 1.9 V). Moreover, He et al. developed wrinkled 2D MoS₂ FETs exhibiting an 8.3 × 10^7^ on/off ratio at 0.6% strain—40 × improvement over planar devices [197]. They demonstrated strain-dependent PL enhancement, with A and B exciton intensities increasing by 77% and 11.2% per 1% strain, respectively, while PL peak shifts revealed 30 meV/% bandgap modulation. The wrinkled FETs showed 70% faster response at Vg = 40 V with enhanced photocurrent. This strain-engineering method effectively optimizes optical and electronic properties in 2D materials, offering a viable strategy for high-performance devices.

#### Kirigami-Inspired Engineering in Stretchable Electronics

Kirigami-inspired engineering has recently emerged as a powerful strategy for achieving extreme stretchability and mechanical compliance in flexible electronic systems [198]. Unlike conventional wrinkling-based approaches that rely on elastic mismatch between layered materials, kirigami exploits geometric instabilities to reconfigure two-dimensional layouts into three-dimensional architectures with programmable mechanical responses. By introducing predesigned cuts or slits into thin films, kirigami architectures harness geometry-guided mechanical instabilities under external loading [199]. These cuts concentrate stress and redistribute strain, inducing controlled out-of-plane deformation, rotation, and buckling, which transform global in-plane stretching into structural deformation and mitigate effective strain in active materials. Mechanically, this behavior parallels classical surface instabilities, where strain energy is released through out-of-plane morphological evolution. Moreover, the mechanical response of kirigami systems can be precisely programmed via geometric parameters such as cut shape, orientation, and spacing, analogous to how wrinkle wavelength, amplitude, and hierarchy govern material-driven instabilities [200]. As such, kirigami represents a geometry-controlled extension of surface instability strategies, expanding the design toolbox for high-performance, stretchable electronics.

Most recently, Wang et al. developed a kirigami-structured bioelectronic patch for organ-conformal electro-transfection (POCKET) [201], in which parametric kirigami design enables exceptional conformability to complex organ geometries and approaches the theoretical maximum effective contact area. The four-layer POCKET architecture establishes a nanopore–cell juxtaposition at the tissue–device interface, allowing uniform and spatially controlled electro-perforation while facilitating efficient intracellular transport of therapeutic payloads. The system demonstrates high delivery efficiency and precise spatial control across multiple organs, leading to effective protection against DNA damage and ischemia–reperfusion injury and subsequent restoration of organ function. This work highlights the translational potential of kirigami-enabled, instability-driven bioelectronic platforms for precise therapeutic intervention in anatomically challenging organs.

### Optical Encryption and Anti-Counterfeiting

Counterfeiting poses a serious global threat to economic stability, national security, and public health. Conventional anti-counterfeiting measures, such as watermarks and QR codes, often fall short due to their predictable and easily replicable nature [202–207]. Instability-induced patterns have shown considerable potential in anti-counterfeiting. The biaxial labyrinth wrinkling pattern exhibits strong dependence on the imperfection in the fabrication. Even using identical fabrication process and control parameters, it is almost impossible to obtain two identical labyrinth wrinkling patterns.

This observation has inspired the researchers to use the unique wrinkling pattern in anti-counterfeiting applications. Their intrinsic randomness and structural complexity allow the creation of “mechanical fingerprints” that are physically unclonable and highly secure. Reversible wrinkle systems capable of responding to light, temperature, or chemical stimuli can display or conceal optical and fluorescent features on demand, adding an extra layer of protection for packaging, labeling, and document verification. The integration of deep learning-based recognition with distinctive wrinkle patterns has further strengthened their reliability and scalability in practical security applications [208].

#### Anti-Counterfeiting Based on Artificial Fingerprint

By analyzing the characteristic features extracted from each wrinkling patterns, significant heterogeneity among these wrinkled microparticles was found, which inspires the researchers that the wrinkling patterns could be used as “artificial microfingerprints” for anti-counterfeiting purpose. Permanent wrinkling offers security through complex wrinkle patterns, whereas dynamic wrinkling enables reversible appearance and disappearance of wrinkles, further boosting security [209, 210].

**Fig. 6:**
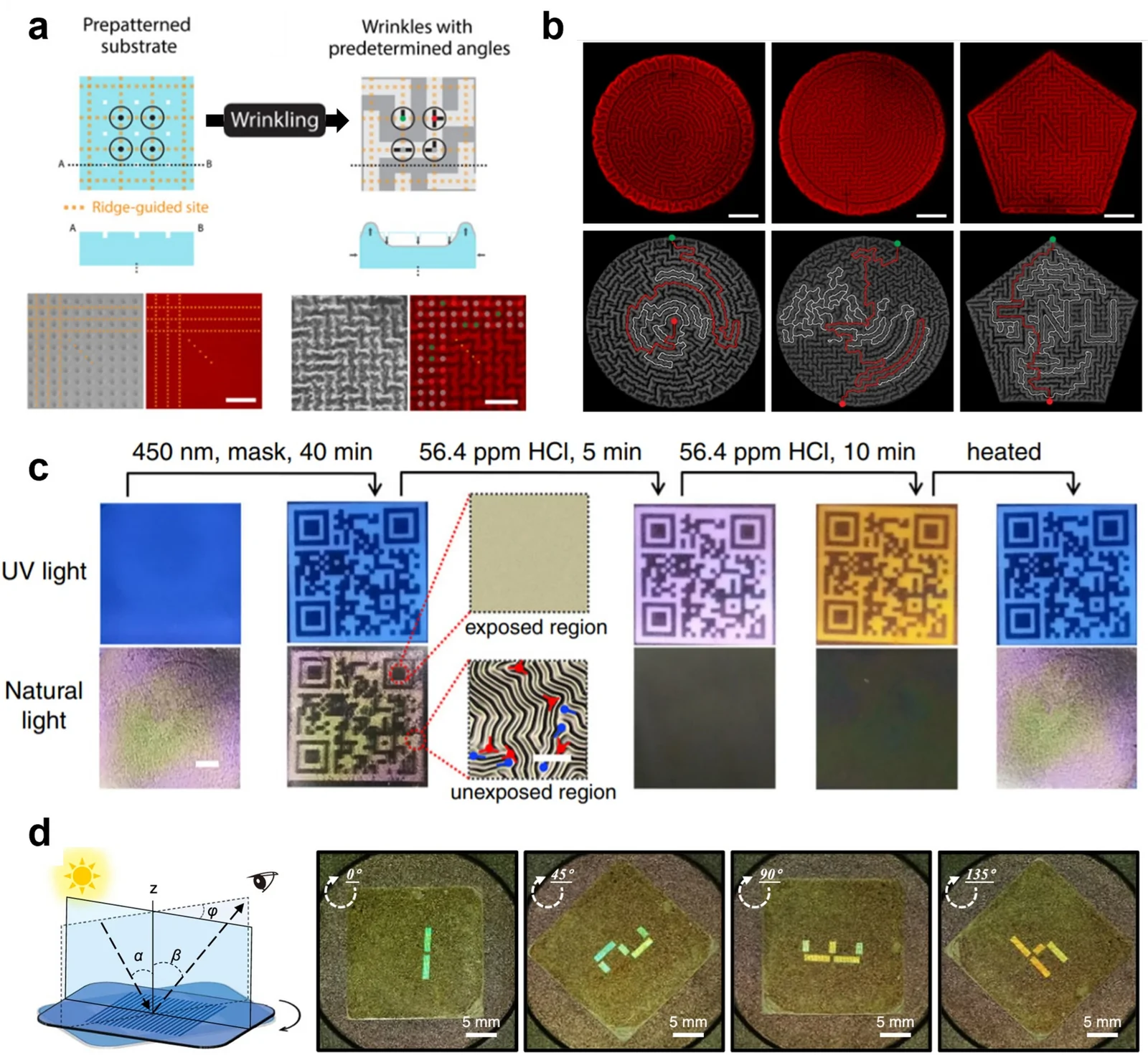
**a** Mechanism for controlling the wrinkling pattern through patterned substrate. **b** Generated wrinkling labyrinths. Reproduced from reference [212]. **c** Flat and QR code shaped fluorescent pattern after undergoing HCl and thermal treatment. Reproduced from reference [214]. **d** Optical images of the wrinkled PAA film at different rotation angles φ (0°, 45°, 90°, and 135°). Reproduced from reference [221]

Bae et al. fabricated microparticles with self-generated random wrinkle patterns similar to human fingerprints [211]. They then conducted the conventional fingerprint reading that detects major features of fingerprint patterns, called “minutiae”, where two representative types are ridge ending and bifurcation. Hundreds of artificial microfingerprints were examined in order to verify their uniqueness. By prepatterning the substrate with a groove array, Bae el al. further demonstrated the location of the ridge ending in the wrinkling patterns could be precisely controlled through a patterned substrate but the bifurcations were still random (Fig. 6a) [212]. By designing the arrangement of the groove arrays, they fabricated the maze with different tessellations, including orthogonal, sigma and theta shapes (Fig. 6b).

Xie et al. fabricated the NIR-driven dynamic wrinkles using the shape memory surface, which could be used as dynamic biomimetic fingerprints [213]. The artificial fingerprints based on dynamic wrinkles could be hidden until exposed to NIR, further improving the security level. The dynamic wrinkles in response to NIR maintain a nearly identical topography during the cycles of erasure and regeneration. Ma et al. fabricated a reversible and multi-responsible wrinkling pattern with simultaneously fluorescence, based on a supramolecular network containing P4VP-nBA-S and DSP-OH [214]. Both the fluorescence and wrinkled topography could be orthogonally modulated by the visible light-triggered isomerization of DSP-OH or acid. Acid-induced protonation of pyridines can dynamically regulate the cross-linking of the skin layer through hydrogen bonding, and the fluorescence of DSP-OH. The authors demonstrated the applications of the dynamic wrinkles with fluorescence in anti-counterfeiting. As shown in Fig. 6c, on selective irradiation with 450 nm visible light or acid treatment, the wrinkles could be generated and flatten reversibly and the fluorescence could be tuned from blue to orange-red.

Ma et al. developed a cost-effective and durable anti-counterfeiting system using physical unclonable function (PUF) labels [215]. The system combines surface wrinkles and phase separation in block copolymers (BCPs) to create hierarchical structures with unique microwrinkles and nanolamellar patterns, resembling human fingerprints. These labels offer an information density 10^10^ times higher than fingerprints and exhibit high bit uniformity, uniqueness, and reliability. The labels' fluorescence changes and 3D wrinkle dimensions provide added security. They also developed a deep learning-based authentication pipeline with nearly 100% accuracy, demonstrating its robustness in real-world scenarios. The PUF labels can be easily applied to various materials and offer excellent environmental resistance, providing a secure and reliable anti-counterfeiting solution. Zhu et al. proposed a fabrication method to create dynamic hierarchical surface wrinkles using a bilayer wrinkling system, which holds potential for anti-counterfeiting applications [21]. The system consists of a thin, rigid skin film made from gelatin mixed with polystyrene (PS) particles, and a soft, thick polydimethylsiloxane substrate. By exploiting the buckling deformation induced by stress instability in the bilayer system, hierarchical surface wrinkles with randomly distributed PS microparticles are formed. By controlling environmental humidity and utilizing the differing sensitivities of the high-modulus exposed regions and the softer, unexposed areas, various dynamic hierarchical structures can be generated.

Most recently, Ma et al. introduced an innovative 2D/3D anti-counterfeiting platform based on anthracene-functionalized poly(styrene-butadiene-styrene) (SBS-CAN) [216], which integrates physically unclonable self-wrinkling patterns, fluorescence, and shape memory functionality. By leveraging the UV-induced dimerization of anthracene units combined with mechanical prestretching, the system enables the generation of 2D codes encoded with both wrinkled surface textures and fluorescence signals—customizable to suit specific security needs. In a further step, 3D structures formed via shape memory effects can be employed to conceal or encrypt the 2D information, significantly raising the security threshold and reducing the risk of duplication based solely on surface morphology or luminescence. This programmable dual-mode (2D/3D) system offers clear advantages over conventional fluorescent wrinkle tags or shape memory labels, representing a highly versatile and intelligent solution for high-security information storage and anti-counterfeiting applications.

#### Anti-Counterfeiting Based on Optical Encryption

At micro- and nanoscales, surface instability patterns interact strongly with light, producing rich optical effects including diffraction, interference, and structural coloration [217–220]. The optical response of wrinkled surfaces is inherently sensitive to subtle variations in pattern morphology, enabling a large design space for generating distinctive and controllable photonic signatures. Building upon these optical properties, surface wrinkling has gained increasing attention as a promising route for optical encoding. By encoding information into the spatial arrangement, orientation, or hierarchical organization of wrinkle patterns, unique optical “fingerprints” can be generated, which are difficult to replicate without access to the exact fabrication parameters.

Zhong et al. proposed a novel wrinkling strategy that combines spatial stress modulation and material integration to realize advanced optical anti-counterfeiting [221]. The controlled diffusion of residual solvent redistributes internal stress, guiding the formation of well-ordered wrinkle patterns. This method enables the encoding and concealment of up to eight distinct switchable images within a single location (Fig. 6d). These images can be visually read without the aid of specialized equipment and without mutual interference (crosstalk). Wen et al. introduced a versatile method for generating dynamic microwrinkles on liquid crystal elastomer (LCE) surfaces [222]. Their approach involves creating micron-scale surface wrinkles on anthracene-functionalized LCE (AnLCE) films through a combination of UV-induced gradient cross-linking followed by mechanical stretching and release (UV-SR). These wrinkles exhibit reversible behavior: upon heating, the LC undergoes a phase transition to an isotropic state, erasing the surface patterns; the wrinkles can then be re-established by repeating the stretching-releasing cycle. This dynamic reversibility, coupled with tunable optical responses under ambient, UV, and polarized light, enables AnLCE films to function as multimodal and reconfigurable display platforms for information decryption and encryption images via wrinkling.

### Tunable Wettability

Switchable (tunable) wettability is of broad industrial significance because it allows surfaces to dynamically tailor liquid–solid interactions, providing on‑demand control over fluid transport, droplet actuation, and surface decontamination. In coatings and self‑cleaning architectures, reversible hydrophobic–hydrophilic transitions underpin anti‑fouling, anti‑icing, and rapid cleaning. In microfluidic platforms and printing technologies, wetting modulation enables deterministic liquid patterning and precise droplet routing, improving throughput and registration accuracy. Beyond these domains, stimuli‑responsive wetting enhances oil–water separation, biomedical interfaces, and heat‑transfer systems by adapting interfacial properties to the local environment. Collectively, this adaptive behavior delivers capabilities unattainable with static surfaces and expands the operational window and efficiency of numerous industrial processes.

Surface wetting property depends on both the surface morphology and the surface chemistry. As an efficient method to generate surface nanostructures, the surface instability has been used to fabricate the structured surface with unique wetting properties [223, 224]. Through surface instability morphologies and suitable chemical modifications [172, 225–227], materials with superhydrophobic characteristics can be obtained, exhibiting high durability, adaptive deformation and rapid droplet mobility. Directional wetting and controllable liquid transport have been achieved using anisotropic wrinkles and gradient surface chemistries [228–230], which are particularly useful for water collection, fog harvesting, and microfluidic control.

#### Superhydrophobic Wetting

**Fig. 7:**
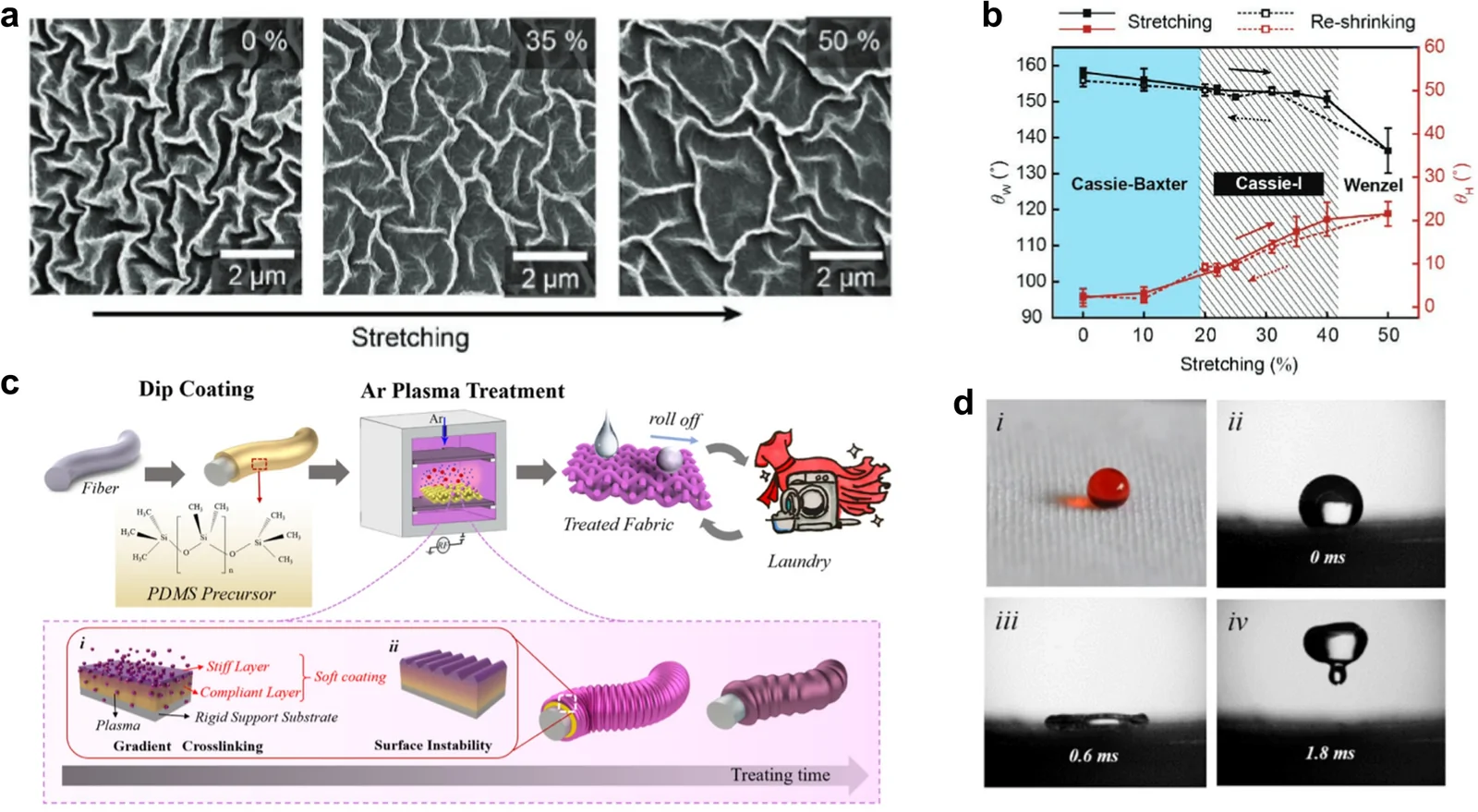
**a** SEM images of the nanoridges during the stretching process at different amounts of stretching. **b** Contact angle (*θ*_*W*_) and hysteresis (*θ*_*H*_) measurements during the reversible topographical transformation with increasing strain up to 50%. Reproduced from reference [231]. **c** Schematic illustrations for the fabrication of superhydrophobic fabrics and formation of wrinkled PDMS on fibers via Ar plasma treatment. **d** Dyed water droplet on the superhydrophobic fabric surface (i) and selected time sequence images of the water droplet impacting the fabric surface (ii − iv). Reproduced from reference [62]

Superhydrophobicity resulting from the Cassie–Baxter wetting state has many fascinating features that has attracted great attentions. Recently, Lee et al. reported a polymeric nanostructure with dynamically tunable wetting properties based on the self-organized nanoridges [231]. The nanoridges with centimeter-scale areas, which were generated by strain relief of fluoropolymer films on polyolefin substrates, have a high aspect ratio greater than four (Fig. 6a). Previously the aspect ratio of wrinkles was limited to be less than one due to the strain localization and subsequent transition to period-double modes. Delamination, on the other hand, has higher aspect ratio but is less uniform. Combined with the surface SF_6_-etching, the nanostructured surface could reversibly transit between the multiple wetting states: Wenzel, Cassie–Baxter, and Cassie-impregnating states in a programmable manner by cyclic stretching and reshrinking (Fig. 7a, b).

Inspired by the earthworm, Xu et al. fabricated an ultradurable superhydrophobic fabrics using the superhydrophobic fibers via Ar plasma treatment as shown in Fig. 7c [62]. The fibers in the fabric were covered by soft wrinkled polymer as the nanostructures for Cassie–Baxter wetting states (Fig. 7d). At the same time, the adaptive wrinkled skin could release stress making the superhydrophobic fabric extraordinary durable. Yu et al. presented a strategy to create ultradurable superamphiphobic fabrics with hierarchical wrinkles, inspired by the deformation adaptability of snakeskin [232]. By infusing perfluorooctyltriethoxysilane (FOS) into a wet chemical and vapor polymerization process, a soft, wrinkled poly-FOS surface is formed. This snakeskin-like texture, combined with high fluorine density, provides the fabric with exceptional water and oil resistance, as well as durability against washing, rubbing, and harsh chemicals.

Hierarchical structures with both nano- and microfeatures were found to better support the Cassie–Baxter wetting states, thus attracted great attention in recent years [233]. Jung et al. used the self-similar hierarchical wrinkles to tune the receding contact angle from 2° to 30° by controlling the wavelength of the hierarchical wrinkles [234]. The hierarchical wrinkles were formed by strain relief of MoS_2_ film on PS substrate. They showed that tunable wettability via surface wrinkling could enhance the hydrogen evolution reaction perfection of the MoS_2_. Chen et al. created robust hierarchically wrinkled nanoporous polytetrafluoroethene (PTFE) surfaces composed of nanoparticles on PTFE wrinkles [235]. By combining the hierarchical interfacial structure and chemical composition, extremely high water repellence with contact angle of ~ 167° and rolling-off angle less than 5° was achieved.

#### Anisotropic Wetting

For decades anisotropic wetting, which could be achieved by either heterogeneous chemical patterning or anisotropic surface morphology, attracted researchers’ interest due to its potential application in directional water transport and water collection [9, 225–228, 230, 236]. Surface instability has been harnessed to generate structured surface with anisotropic wetting property [145, 237, 238].

Rhee et al. fabricated the crack-free soft fluoropolymer layer with uniform wrinkles on PDMS substrates [229]. By applying tensile strain to initial prestretched strain, the 1D wrinkles were flatten. Further increasing the tensile strain leads to the formation of 1D wrinkles perpendicular to the initial direction, due to the compressive stress from Poisson’s effect. Anisotropic wetting was found on the 1D wrinkles with the water spreading along the wrinkle orientation and confined perpendicular to the wrinkle orientation. During the transformation of structural morphology in response to mechanical strain, the water spreading orientation could be tuned. In order to further enhance the wetting anisotropy, Chai et al. prepared a double-gradient wrinkled structure by constructing the structure-gradient pillar arrays on a wrinkled surface and subsequently making chemical gradient by oxygen plasma treatment [239]. Under the synergistic effect of the anisotropic structure and chemical gradient, strong anisotropic wetting was observed and the spreading length on the flexible structure could be dynamically regulated in a broad range by external strain. Kwon et al. generated a structured surface using line patterned surface combining wrinkles and cracks [145]. Tunable anisotropy and orientation of liquid wetting were investigated by the authors.

The formation of the anisotropic wetting is due to the contact line pinning from the surface structure. Lin et al. analyzed the contact line motion and quantitatively explained the observed small degree of anisotropic wetting on the multiscale self-similar hierarchical wrinkled surface which is composed of short-period wrinkles on the long-period wrinkles with the same orientation [240]. The orthogonal cracks were also observed accompanying the hierarchical wrinkles. The wetting state is confirmed as the Wenzel state using confocal imaging technique. They calculated the energy barriers of the three-phase contact line pinning from both large wrinkles and small wrinkles and predicted the anisotropic contact angles based on a thermodynamic model.

### Biomedical Application

Surface instability is widely observed in living tissues, and its mechanically guided patterns play an essential role in many biomedical processes. From cortical folding in the brain to the wrinkled morphology of mucosa and epithelial layers, these patterns support key biomechanical and physiological functions. For example, the development of brain cortex greatly enhances the intellectual capacity [241–243], the growth-induced wrinkled membrane of cells provides excess surface area to support deformation [244, 245], and the villi of small intestine facilitate the absorption of nutrient among intestinal cells [246, 247].

Recreating surface instabilities in synthetic materials provides an effective pathway to probe tissue morphogenesis and to design structures that emulate natural organ function. Youn et al. introduced an in vitro tissue-scale epithelial bilayer folding model that combines an epithelium and extracellular matrix (ECM) hydrogel to mimic in vivo folding patterns [248]. Unlike animal models, this system allows real-time observation and independent control of experimental parameters, providing a valuable tool for investigating the mechanisms behind epithelial folding in developmental biology and tissue engineering. These advances underscore the growing importance of surface instability as a foundational strategy in the development of biomedical models, tissue engineering platforms, and artificial organ systems.

**Fig. 8:**
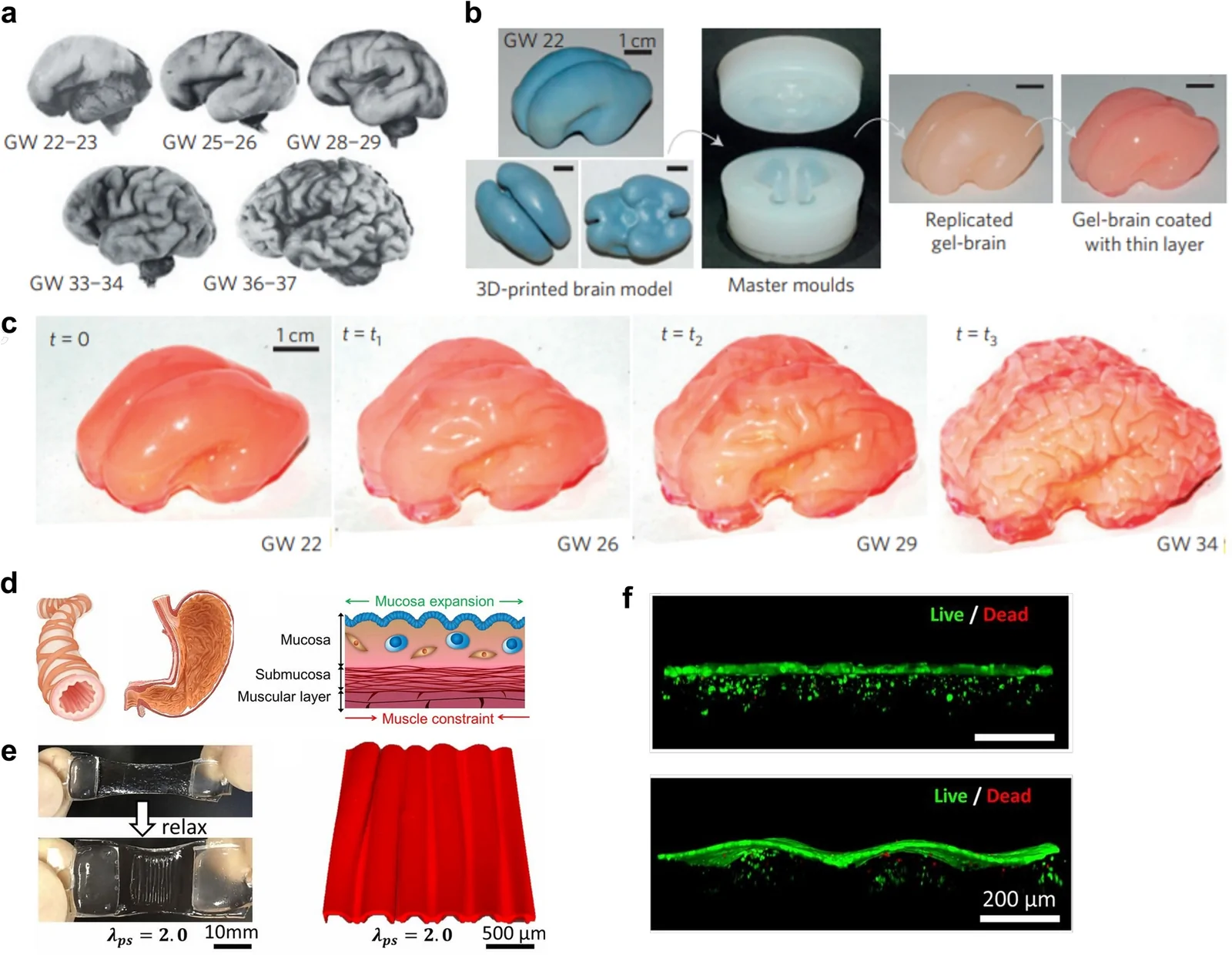
**a** Gyrification of the human brain during the latter half of gestation. **b** A 3D printed model of the brain is produced from a 3D MRI image of a smooth fetal brain. To mimic the constrained growth of the cortex, a replicated gel-brain (white matter) is coated with a thin layer of gel (cortex) that swells by absorbing a solvent (hexanes) over time t (t_1_ ≈4 min, t_2_ ≈9 min, t3 ≈16 min). **c** Layered gel progressively evolves into a complex pattern of sulci and gyri during the swelling process. Reproduced from reference [251]. **d** Left: Schematic showing the uniaxial patterns developed on the lumen of typical tubular structures such as bronchus and the biaxial patterns developed on the lumen found in the digestive, respiratory, or reproductive tracts. **e** Left: GelMA-coated prestretched tough hydrogel before and after uniaxial relaxation. Right: Top view of Rhodamine-B–labeled GelMA (3D confocal reconstruction) showing the surface morphology. **f** Three-dimensional confocal reconstruction of coculture of Ishikawa cells and tHESCs stained for live and dead cells. Reproduce from reference [129]

Hydrogels with hydrophilic porous networks are promising materials for nascent applications in artificial organs due to their high water content, biocompatibility and tunable mechanical properties [249, 250]. Generating wrinkling patterns on the hydrogel surface renders hydrogels to biomimic the complex morphology or shape of tissues and organs. Several researchers have developed the versatility and adaptability of wrinkling hydrogels system for the fabrication of biomimetic tissue and artificial organs. For example, Tallinen et al. mimicked cortical growth in fetal brain by swelling a 3D printed brain model made of layered gel (Fig. 8a, b) [251]. The strain mismatch between the outer layer and core induced the growth and form of folds similar to the sulci and gyri on the brain (Fig. 8c). By modeling brain as soft tissue, they showed that mechanical instability is the main factor that determined the size and morphology of such cortical convolutions.

The surfaces of many hollow or tubular tissues/organs in our digestive, respiratory, or reproductive tracts are covered by mucosa with folded patterns (Fig. 8d). Understanding this formation process of these folded patterns will facilitate the engineering of mucosa in various tissues/organs. Li et al. studied the mechanics of surface wrinkling morphology of mucosa [252]. The wrinkling patterns are induced by surface instability of the mucosa under compression due to constrained growth. Later, Chan et al. demonstrated a simple method to fabricate folding artificial mucosa via surface instability of cell-laden hydrogel biofilms [129]. A hydrogel film of gelatin methacrylate (GelMA) encapsulated with stromal cell line hTERT-immortalized human endometrial stromal cells (tHESCs) is fabricated on the prestretched tough hydrogel substrate made of interpenetrating polymer networks of polyacrylamide (PAAm) and alginate. A layer of epithelial cell (Ishikawa human endometrial adenocarcinoma cells) is cultured on top of the GelMA film. The relaxation of uniaxially or biaxially prestretched substrate applies a uniaxial or biaxial compressive strain, respectively, on the cell-laden hydrogel film to generate wrinkles as shown in Fig. 8e. Both the flat and folded coculture systems exhibit excellent viability of tHESCs and Ishikawa cells as evidenced by live–dead staining (Fig. 8f).

## Prospectives

This review has provided a comprehensive and integrated overview of surface instabilities, spanning fundamental mechanisms, fabrication strategies, and emerging applications. By systematically unifying multimode instability phenomena and cross-scale morphological control, it clarifies the intrinsic connections among wrinkling, folding, creasing, and delamination-induced buckling, thereby offering a coherent framework for understanding and designing complex surface morphologies. Beyond mechanistic insights, the review highlights advanced fabrication and design strategies that enable hierarchical and programmable surface architectures from the nanoscale to the microscale. Particular emphasis is placed on frontier applications, including stimuli-responsive interfaces, dynamically tunable wettability, optical encryption and anti-counterfeiting, and biomimetic platforms for biomedical engineering, illustrating the transformative potential of instability-enabled surface engineering.

**Fig. 9:**
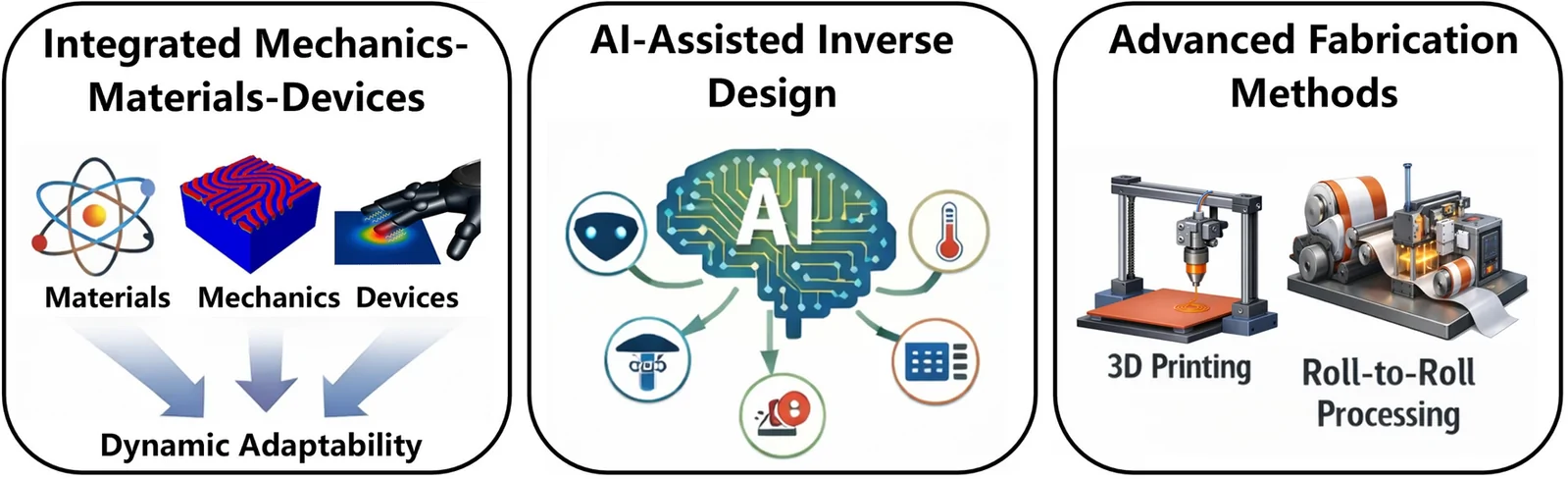
Schematic illustration of key future directions for instability-enabled functional materials and surfaces. The perspective highlights three synergistic directions: (i) an integrated mechanics–materials–devices paradigm, which bridges fundamental in stability theory with device-level applications; (ii) AI-assisted inverse design, enabling a function-driven “performance-to-structure” strategy for exploring optimal instability architectures; and (iii) advanced fabrication methods, including 3D printing and roll-to-roll processing, which provide scalable pathways for translating instability-based designs into practical technologies

Future research directions are anticipated to focus on predictive modeling, multifunctional material systems, and intelligent adaptive surfaces, ultimately accelerating the translation of surface instability concepts into practical technologies. As shown in Fig. 9, the integrated “mechanics–materials–devices” paradigm plays a pivotal role in bridging fundamental instability theory with device-level implementation by enabling dynamic adaptability across multiple length and time scales. Within this framework, mechanical instabilities are no longer treated as static structural outcomes, but as actively exploitable deformation modes whose onset, evolution, and reversibility can be programmed through material selection and mechanical design. AI-assisted inverse design has opened a new design strategy, in which desired end functionalities—whether optical, mechanical, thermal, or electrical—serve as the starting point, and artificial intelligence functions as the core reasoning engine to automatically explore optimal or feasible structural configurations capable of achieving these targets [253, 254]. This approach is particularly suitable for handling complex problems involving material nonlinearity, large geometric deformations, and multiphysics coupling, and has become a research forefront in the design of intelligent surface structures. Combining machine learning-based predictive models with real-time sensing and feedback could further enable autonomous systems with self-adaptive surface reconfiguration capabilities [255]. Furthermore, the integration of instability-based design principles with additive manufacturing, roll-to-roll processing, and hybrid soft–rigid assembly techniques offer practical routes toward scalable and industrially viable implementation.

Several challenges still limit the broader implementation of instability-based design. Achieving scalable and uniform control of complex surface morphologies over large areas remains difficult, especially when rigid or intricately shaped substrates are involved. Many existing fabrication approaches still rely on mechanical prestraining, which constrains versatility and reproducibility. In addition, coordinating predictable responses under multiple external stimuli is challenging, as different activation mechanisms often interact in a nonlinear and poorly controlled manner. Long-term structural and functional stability under cyclic loading, environmental exposure, and repeated actuation also remains insufficiently understood. Addressing these issues will require reconfigurable, stimuli-responsive systems that can be activated by electrical, magnetic, thermal, or biochemical cues, enabling dynamic and programmable control of surface morphology and paving the way for multifunctional, adaptive devices. Several cross-disciplinary strategies may be pursued: (i) developing material systems with intrinsically programmable or self-regulating mechanical properties to reduce reliance on mechanical prestraining; (ii) integrating multiphysics modeling with real-time characterization to guide the coupled design of mechanics, materials, and stimuli fields; (iii) incorporating advances in soft electronics, smart materials, and bioinspired architectures to enable robust, multifunctional, and adaptive surface systems. Together, these approaches may help bridge microscale instability control with macroscale performance and reliability, accelerating the translation of surface instabilities into practical devices.

Looking forward, the continued convergence of mechanics, materials science, soft robotics, and intelligent systems will propel surface instability research into new territories [256]. By addressing current limitations in fabrication, control, and scalability and by leveraging data-driven design and novel material platforms, surface instabilities are poised to become a foundational principle in the development of next-generation adaptive, intelligent, and multifunctional surfaces.
